# (Dimethyl­formamide-κ*O*)(2-hy­droxy­benzoato-κ^2^ *O* ^1^,*O* ^1′^)[tris­(1-methyl-1*H*-benzimidazol-2-ylmethyl-κ*N* ^3^)amine-κ*N*]manganese(II) perchlorate dimethyl­formamide monosolvate

**DOI:** 10.1107/S160053681003610X

**Published:** 2010-09-11

**Authors:** Huilu Wu, Ying Bai, Xingcai Huang, Xuan Meng, Baoliang Qi

**Affiliations:** a School of Chemical and Biological Engineering, Lanzhou Jiaotong University, Lanzhou 730070, People’s Republic of China

## Abstract

In the title complex, [Mn(C_7_H_5_O_3_)(C_27_H_27_N_7_)(C_3_H_7_NO)]ClO_4_·C_3_H_7_NO, the Mn^II^ ion is coordinated in a slightly distorted monocapped trigonal-prismatic geometry. The tris­(1-methyl-1*H*-benzimidazol-2-ylmeth­yl)amine (Mentb) ligand coordinates in a tetra­dentate mode and the coordination is completed by a bis-chelating salicylate ligand and a dimethyl­formamide ligand. The hy­droxy group and the *ortho* H atoms of the salicylate ligand were refined as disordered over two sites with occupancies of 0.581 (8) and 0.419 (8). Both disorder components of the hy­droxy group form intra­molecular O—H⋯O hydrogen bonds.

## Related literature

For the biological activity of benzimidazole compounds, see: Horton *et al.* (2003 ▶). For related structures, see: Wu *et al.* (2005 ▶, 2009 ▶).
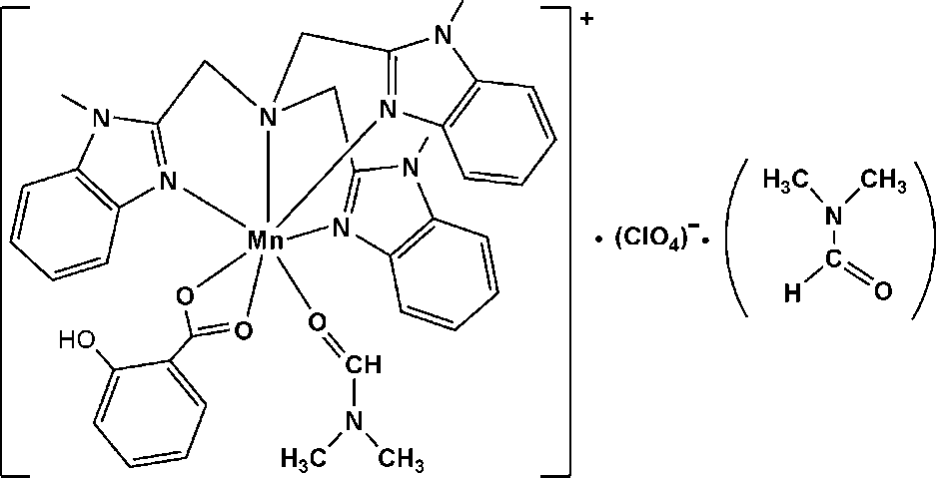


## Experimental

### 

#### Crystal data

[Mn(C_7_H_5_O_3_)(C_27_H_27_N_7_)(C_3_H_7_NO)]ClO_4_·C_3_H_7_NO
*M* *_r_* = 887.25
Triclinic, 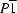

*a* = 12.3689 (13) Å
*b* = 12.4809 (13) Å
*c* = 15.3759 (16) Å
α = 69.925 (1)°
β = 87.926 (1)°
γ = 74.704 (1)°
*V* = 2146.6 (4) Å^3^
*Z* = 2
Mo *K*α radiation
μ = 0.43 mm^−1^
*T* = 296 K
0.38 × 0.36 × 0.32 mm

#### Data collection

Bruker SMART APEX diffractometer
Absorption correction: multi-scan (*SADABS*; Sheldrick, 1996 ▶) *T*_min_ = 0.853, *T*_max_ = 0.874
15941 measured reflections
7890 independent reflections
6197 reflections with *I* > 2σ(*I*)
*R*_int_ = 0.032

#### Refinement

*R*[*F* ^2^ > 2σ(*F* ^2^)] = 0.052
*wR*(*F* ^2^) = 0.163
*S* = 1.10
7890 reflections
560 parameters
16 restraints
H-atom parameters constrained
Δρ_max_ = 0.54 e Å^−3^
Δρ_min_ = −0.57 e Å^−3^

### 

Data collection: *SMART* (Bruker, 2000 ▶); cell refinement: *SAINT* (Bruker, 2000 ▶); data reduction: *SAINT*; program(s) used to solve structure: *SHELXS97* (Sheldrick, 2008 ▶); program(s) used to refine structure: *SHELXL97* (Sheldrick, 2008 ▶); molecular graphics: *PLATON* (Spek, 2009 ▶); software used to prepare material for publication: *SHELXTL* (Sheldrick, 2008 ▶).

## Supplementary Material

Crystal structure: contains datablocks global, I. DOI: 10.1107/S160053681003610X/lh5127sup1.cif

Structure factors: contains datablocks I. DOI: 10.1107/S160053681003610X/lh5127Isup2.hkl

Additional supplementary materials:  crystallographic information; 3D view; checkCIF report

## Figures and Tables

**Table 1:** Hydrogen-bond geometry (Å, °)

*D*—H⋯*A*	*D*—H	H⋯*A*	*D*⋯*A*	*D*—H⋯*A*
O4′—H4′⋯O2	0.82	1.82	2.554 (8)	149
O4—H4⋯O1	0.82	1.79	2.530 (5)	149

**Fig. 1.**
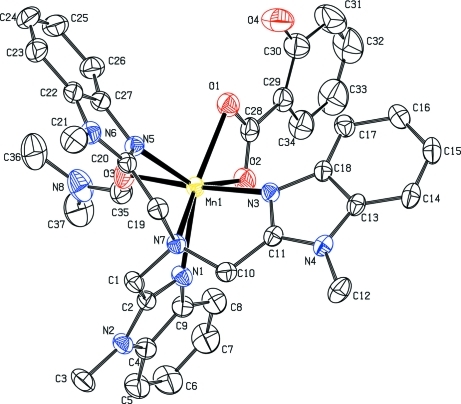
The cation of the title compound with displacement ellipsoides drawn at the 30% probability level. H atoms have been omitted for clarity.

## supplementary crystallographic information

### Comment

We are interested in tris(2-benzimidazolylmethyl)amine and its derivatives and we have previously reported the crystal structure of some related complexes (Wu *et al.*, 2009; Wu *et al.*, 2005). The benzimidazole core is of a wide interest because of its diverse biological activities, and it is well known in medicinal chemistry (Horton *et al.* 2003). The asymmetric unit of the title compound consists of a [Mn(Mentb)(salicylato)(DMF)] cation, a perchlorate anion, and one solvent molecule of DMF. The cation is shown in Fig. 1. The Mn^II^ ion is coordinated in a slightly distorted monocapped trigonal-prismatic geometry. The tris(1-methyl-1*H*-benzimidazol-2-ylmethyl)amine (Mentb) ligand coordinates in a tetradentate mode and the coordination is completed by a bis-chelating salicylato ligand and dimethylformamide ligand. The hydroxy group of the salicylato ligand is disordered over two sites with occupancies 0.581 (8) and 0.419 (8). Both disorder components form intramolecular O-H···O hydrogen bonds.

### Experimental

To a stirred solution of Mentb (0.5 mmol) in hot methanol (20 ml), Mn(ClO_4_)_2_.6 H_2_O (0.5 mmol) and a solution of Na(salicylato) (0.5 mmol) in methanol (5 ml) was added. A colorless microcrystalline precipitate was produced and collected by filtration. After drying in air the colorless product was redissolved in DMF/methanol (1:1) and filtered. Light-yellow block-shaped crystals suitable for X-ray diffraction studies were obtained by vapor diffusion of diethyl ether into the filtrate for 4 weeks at room temperature. (Yield 0.12 g, 67%). Elemental analysis found: C, 54.09%; H, 5.59%; N, 14.13%; calcd. for C40 H46 O9 N9 Mn Cl: C, 54.15%; H, 5.23%; N, 14.21%.

### Refinement

All H atoms were geometrically positioned and refined using a riding-model approximation with C—H distances from 0.93 to 0.97 Å and O—H = 0.82 Å, and with *U*_iso_(H) = 1.2 *U*_eq_(C) or *U*_iso_(H) = 1.5 *U*_eq_(C_methyl_,*O*). The hydroxy group and the ortho-H atom of the salicylato ligand were refined as disordered over two sites with occupancies 0.581 (8) and 0.419 (8). In the final refinement cycles restraints were applied to some anisotropic displacement parameters (EADP, SIMU and DELU instructions in *SHELXL97*, Sheldrick, 2008) to obtain more sensible values i.e. EADP C30 C30' C34 C34', SIMU N8 C36 C37, DELU MN1 O1 O2 C36 C37.

### Crystal data

**Table:**

[Mn(C_7_H_5_O_3_)(C_27_H_27_N_7_)(C_3_H_7_NO)]ClO_4_·C_3_H_7_NO	*Z* = 2
*M**_r_* = 887.25	*F*(000) = 926
Triclinic, *P*1	*D*_x_ = 1.373 Mg m^−^^3^
Hall symbol: -P 1	Mo *K*α radiation, λ = 0.71073 Å
*a* = 12.3689 (13) Å	Cell parameters from 9784 reflections
*b* = 12.4809 (13) Å	θ = 1.7–27.7°
*c* = 15.3759 (16) Å	µ = 0.43 mm^−^^1^
α = 69.925 (1)°	*T* = 296 K
β = 87.926 (1)°	Block, light-yellow
γ = 74.704 (1)°	0.38 × 0.36 × 0.32 mm
*V* = 2146.6 (4) Å^3^	

### Data collection

**Table:**

Bruker SMART APEX diffractometer	7890 independent reflections
Radiation source: fine-focus sealed tube	6197 reflections with *I* > 2σ(*I*)
graphite	*R*_int_ = 0.032
ω scans	θ_max_ = 25.5°, θ_min_ = 1.7°
Absorption correction: multi-scan (*SADABS*; Sheldrick, 1996)	*h* = −13→14
*T*_min_ = 0.853, *T*_max_ = 0.874	*k* = −15→15
15941 measured reflections	*l* = −18→18

### Refinement

**Table:**

Refinement on *F*^2^	Primary atom site location: structure-invariant direct methods
Least-squares matrix: full	Secondary atom site location: difference Fourier map
*R*[*F*^2^ > 2σ(*F*^2^)] = 0.052	Hydrogen site location: inferred from neighbouring sites
*wR*(*F*^2^) = 0.163	H-atom parameters constrained
*S* = 1.10	*w* = 1/[σ^2^(*F*_o_^2^) + (0.1005*P*)^2^ + 0.2919*P*] where *P* = (*F*_o_^2^ + 2*F*_c_^2^)/3
7890 reflections	(Δ/σ)_max_ = 0.001
560 parameters	Δρ_max_ = 0.54 e Å^−^^3^
16 restraints	Δρ_min_ = −0.57 e Å^−^^3^

### Special details

**Table:**

Geometry. All e.s.d.'s (except the e.s.d. in the dihedral angle between two l.s. planes) are estimated using the full covariance matrix. The cell e.s.d.'s are taken into account individually in the estimation of e.s.d.'s in distances, angles and torsion angles; correlations between e.s.d.'s in cell parameters are only used when they are defined by crystal symmetry. An approximate (isotropic) treatment of cell e.s.d.'s is used for estimating e.s.d.'s involving l.s. planes.
Refinement. Refinement of *F*^2^ against ALL reflections. The weighted *R*-factor *wR* and goodness of fit *S* are based on *F*^2^, conventional *R*-factors *R* are based on *F*, with *F* set to zero for negative *F*^2^. The threshold expression of *F*^2^ > σ(*F*^2^) is used only for calculating *R*-factors(gt) *etc*. and is not relevant to the choice of reflections for refinement. *R*-factors based on *F*^2^ are statistically about twice as large as those based on *F*, and *R*- factors based on ALL data will be even larger.

### Fractional atomic coordinates and isotropic or equivalent isotropic displacement parameters (Å^2^)

**Table:**

	*x*	*y*	*z*	*U*_iso_*/*U*_eq_	Occ. (<1)
C1	0.7376 (2)	0.8865 (3)	0.89825 (18)	0.0441 (6)	
H1A	0.7582	0.8230	0.9577	0.053*	
H1B	0.7241	0.9614	0.9083	0.053*	
C2	0.8306 (2)	0.8753 (2)	0.83346 (18)	0.0411 (6)	
C3	0.9618 (3)	0.9470 (4)	0.9088 (2)	0.0655 (9)	
H3A	0.8963	0.9830	0.9346	0.098*	
H3B	1.0020	1.0047	0.8791	0.098*	
H3C	1.0094	0.8826	0.9575	0.098*	
C4	0.9896 (2)	0.8843 (3)	0.7675 (2)	0.0495 (7)	
C5	1.0948 (3)	0.8994 (4)	0.7409 (2)	0.0690 (10)	
H5	1.1383	0.9238	0.7746	0.083*	
C6	1.1304 (3)	0.8764 (4)	0.6627 (3)	0.0844 (12)	
H6	1.2001	0.8860	0.6423	0.101*	
C7	1.0658 (3)	0.8387 (4)	0.6119 (3)	0.0794 (11)	
H7	1.0938	0.8236	0.5590	0.095*	
C8	0.9612 (3)	0.8234 (3)	0.6387 (2)	0.0621 (9)	
H8	0.9178	0.7993	0.6048	0.075*	
C9	0.9239 (2)	0.8458 (3)	0.71914 (19)	0.0472 (7)	
C10	0.5833 (2)	0.9924 (2)	0.78241 (19)	0.0441 (6)	
H10A	0.6400	1.0316	0.7532	0.053*	
H10B	0.5308	1.0443	0.8086	0.053*	
C11	0.5227 (2)	0.9678 (2)	0.71201 (17)	0.0397 (6)	
C12	0.4095 (3)	1.1790 (3)	0.6354 (2)	0.0643 (9)	
H12A	0.3289	1.2033	0.6337	0.096*	
H12B	0.4349	1.2245	0.5784	0.096*	
H12C	0.4399	1.1918	0.6864	0.096*	
C13	0.4097 (2)	0.9988 (2)	0.59389 (17)	0.0423 (6)	
C14	0.3347 (2)	1.0450 (3)	0.51700 (19)	0.0522 (7)	
H14	0.2986	1.1254	0.4923	0.063*	
C15	0.3163 (3)	0.9653 (3)	0.47902 (19)	0.0561 (8)	
H15	0.2665	0.9923	0.4274	0.067*	
C16	0.3711 (3)	0.8456 (3)	0.5166 (2)	0.0556 (8)	
H16	0.3570	0.7943	0.4894	0.067*	
C17	0.4458 (2)	0.8004 (3)	0.59322 (19)	0.0488 (7)	
H17	0.4810	0.7198	0.6183	0.059*	
C18	0.4666 (2)	0.8785 (2)	0.63128 (16)	0.0399 (6)	
C19	0.5560 (2)	0.8445 (3)	0.92604 (18)	0.0446 (6)	
H19A	0.4808	0.8721	0.8969	0.053*	
H19B	0.5567	0.8798	0.9732	0.053*	
C20	0.5877 (2)	0.7134 (2)	0.96938 (17)	0.0411 (6)	
C21	0.4895 (3)	0.7020 (4)	1.1177 (2)	0.0723 (10)	
H21A	0.5382	0.7067	1.1627	0.108*	
H21B	0.4425	0.6520	1.1488	0.108*	
H21C	0.4434	0.7798	1.0838	0.108*	
C22	0.5982 (3)	0.5329 (3)	1.06591 (19)	0.0520 (7)	
C23	0.5914 (3)	0.4311 (4)	1.1384 (2)	0.0670 (10)	
H23	0.5535	0.4350	1.1910	0.080*	
C24	0.6433 (3)	0.3254 (4)	1.1283 (3)	0.0754 (11)	
H24	0.6410	0.2556	1.1755	0.091*	
C25	0.6995 (3)	0.3191 (3)	1.0496 (3)	0.0720 (10)	
H25	0.7337	0.2454	1.0452	0.086*	
C26	0.7057 (3)	0.4213 (3)	0.9769 (2)	0.0602 (8)	
H26	0.7430	0.4171	0.9241	0.072*	
C27	0.6544 (2)	0.5283 (3)	0.98641 (19)	0.0466 (7)	
C28	0.7141 (3)	0.6110 (3)	0.66229 (19)	0.0504 (7)	
C29	0.7189 (3)	0.5550 (3)	0.5908 (2)	0.0539 (8)	
C30	0.6454 (4)	0.4871 (3)	0.5915 (3)	0.0749 (11)	0.581 (8)
C30'	0.6454 (4)	0.4871 (3)	0.5915 (3)	0.0749 (11)	0.419 (8)
H30'	0.5962	0.4728	0.6387	0.090*	0.419 (8)
C31	0.6466 (5)	0.4406 (4)	0.5206 (4)	0.1059 (17)	
H31	0.5959	0.3979	0.5189	0.127*	
C32	0.7223 (6)	0.4582 (5)	0.4537 (4)	0.117 (2)	
H32	0.7225	0.4270	0.4068	0.141*	
C33	0.7980 (5)	0.5207 (5)	0.4542 (3)	0.1068 (17)	
H33	0.8507	0.5295	0.4093	0.128*	
C34	0.7953 (4)	0.5708 (4)	0.5220 (2)	0.0740 (11)	0.581 (8)
H34	0.8450	0.6154	0.5217	0.089*	0.581 (8)
C34'	0.7953 (4)	0.5708 (4)	0.5220 (2)	0.0740 (11)	0.419 (8)
C35	0.9528 (3)	0.5539 (3)	0.8186 (2)	0.0605 (8)	
H35	0.9588	0.6037	0.7588	0.073*	
C36	1.0375 (4)	0.3827 (4)	0.9514 (3)	0.0990 (11)	
H36A	0.9634	0.4017	0.9727	0.148*	
H36B	1.0911	0.3839	0.9945	0.148*	
H36C	1.0544	0.3053	0.9468	0.148*	
C37	1.1502 (4)	0.4571 (5)	0.8161 (4)	0.1028 (11)	
H37A	1.1690	0.3842	0.8038	0.154*	
H37B	1.2075	0.4559	0.8571	0.154*	
H37C	1.1447	0.5222	0.7589	0.154*	
C38	0.8240 (8)	0.1803 (7)	0.6898 (7)	0.186 (5)	
H38	0.8428	0.1515	0.6413	0.223*	
C39	0.8823 (10)	0.2884 (7)	0.7733 (7)	0.221 (6)	
H39A	0.8069	0.3022	0.7931	0.332*	
H39B	0.9014	0.3624	0.7480	0.332*	
H39C	0.9332	0.2368	0.8254	0.332*	
C40	0.9828 (8)	0.2465 (10)	0.6515 (7)	0.248 (7)	
H40A	0.9865	0.2017	0.6112	0.373*	
H40B	1.0505	0.2163	0.6908	0.373*	
H40C	0.9746	0.3281	0.6150	0.373*	
Cl	0.25816 (7)	0.06650 (8)	0.87509 (6)	0.0620 (2)	
Mn1	0.69993 (3)	0.72415 (3)	0.78373 (2)	0.03771 (15)	
N1	0.82321 (19)	0.8399 (2)	0.76340 (15)	0.0446 (5)	
N2	0.9278 (2)	0.9023 (2)	0.84077 (16)	0.0463 (6)	
N3	0.53926 (18)	0.86162 (19)	0.70643 (14)	0.0387 (5)	
N4	0.44709 (19)	1.05312 (19)	0.64708 (15)	0.0430 (5)	
N5	0.64677 (19)	0.6439 (2)	0.92610 (14)	0.0423 (5)	
N6	0.5571 (2)	0.6524 (2)	1.05338 (15)	0.0497 (6)	
N7	0.63642 (18)	0.88007 (19)	0.85613 (14)	0.0383 (5)	
N8	1.0432 (3)	0.4710 (3)	0.8591 (3)	0.0839 (8)	
N9	0.8900 (4)	0.2366 (4)	0.7067 (4)	0.1084 (15)	
O1	0.6542 (2)	0.58442 (19)	0.73067 (14)	0.0578 (5)	
O2	0.76700 (19)	0.6868 (2)	0.65364 (14)	0.0567 (5)	
O3	0.86189 (19)	0.5726 (2)	0.85104 (15)	0.0619 (6)	
O4	0.5786 (5)	0.4636 (5)	0.6574 (4)	0.089 (2)	0.581 (8)
H4	0.5827	0.5000	0.6921	0.134*	0.581 (8)
O4'	0.8645 (8)	0.6282 (9)	0.5211 (7)	0.113 (4)	0.419 (8)
H4'	0.8451	0.6670	0.5553	0.170*	0.419 (8)
O5	0.3371 (3)	0.1263 (3)	0.8814 (2)	0.1019 (10)	
O6	0.2796 (3)	0.0238 (4)	0.8013 (2)	0.1131 (12)	
O7	0.2718 (4)	−0.0358 (3)	0.9587 (2)	0.1162 (12)	
O8	0.1490 (3)	0.1332 (4)	0.8762 (3)	0.1210 (13)	
O9	0.7465 (5)	0.1614 (5)	0.7262 (6)	0.231 (4)	

### Atomic displacement parameters (Å^2^)

**Table:**

	*U*^11^	*U*^22^	*U*^33^	*U*^12^	*U*^13^	*U*^23^
C1	0.0370 (15)	0.0586 (17)	0.0417 (13)	−0.0167 (13)	0.0033 (11)	−0.0207 (12)
C2	0.0369 (15)	0.0447 (15)	0.0409 (13)	−0.0135 (11)	0.0001 (10)	−0.0116 (11)
C3	0.061 (2)	0.091 (3)	0.0614 (19)	−0.0396 (19)	−0.0005 (15)	−0.0326 (18)
C4	0.0390 (16)	0.0599 (18)	0.0479 (15)	−0.0193 (13)	0.0044 (12)	−0.0123 (13)
C5	0.0460 (19)	0.098 (3)	0.067 (2)	−0.0360 (19)	0.0088 (15)	−0.0218 (19)
C6	0.053 (2)	0.129 (4)	0.080 (2)	−0.046 (2)	0.0261 (19)	−0.034 (2)
C7	0.065 (2)	0.116 (3)	0.064 (2)	−0.032 (2)	0.0289 (18)	−0.035 (2)
C8	0.053 (2)	0.084 (2)	0.0551 (17)	−0.0226 (17)	0.0146 (14)	−0.0290 (16)
C9	0.0381 (16)	0.0556 (17)	0.0465 (14)	−0.0159 (13)	0.0072 (11)	−0.0137 (12)
C10	0.0420 (16)	0.0414 (15)	0.0489 (14)	−0.0115 (12)	0.0029 (11)	−0.0156 (12)
C11	0.0359 (14)	0.0409 (14)	0.0375 (12)	−0.0076 (11)	0.0063 (10)	−0.0099 (10)
C12	0.076 (2)	0.0404 (16)	0.0592 (18)	−0.0012 (15)	0.0006 (16)	−0.0070 (14)
C13	0.0368 (15)	0.0477 (15)	0.0332 (12)	−0.0090 (12)	0.0065 (10)	−0.0047 (11)
C14	0.0395 (16)	0.0588 (18)	0.0404 (14)	−0.0049 (13)	0.0003 (11)	−0.0011 (13)
C15	0.0432 (17)	0.080 (2)	0.0360 (13)	−0.0149 (15)	−0.0015 (12)	−0.0094 (14)
C16	0.0479 (18)	0.075 (2)	0.0497 (16)	−0.0200 (16)	0.0023 (13)	−0.0267 (15)
C17	0.0408 (16)	0.0550 (17)	0.0489 (15)	−0.0116 (13)	0.0001 (12)	−0.0168 (13)
C18	0.0321 (14)	0.0483 (15)	0.0339 (12)	−0.0108 (11)	0.0030 (10)	−0.0076 (11)
C19	0.0353 (15)	0.0556 (17)	0.0442 (14)	−0.0126 (12)	0.0076 (11)	−0.0192 (12)
C20	0.0321 (14)	0.0537 (16)	0.0380 (13)	−0.0149 (12)	0.0031 (10)	−0.0141 (11)
C21	0.075 (3)	0.097 (3)	0.0548 (18)	−0.036 (2)	0.0285 (17)	−0.0307 (18)
C22	0.0440 (17)	0.064 (2)	0.0435 (14)	−0.0255 (14)	−0.0028 (12)	−0.0049 (13)
C23	0.060 (2)	0.078 (3)	0.0538 (18)	−0.0344 (19)	0.0000 (15)	0.0004 (16)
C24	0.071 (3)	0.063 (2)	0.073 (2)	−0.035 (2)	−0.0107 (19)	0.0135 (18)
C25	0.064 (2)	0.051 (2)	0.092 (3)	−0.0200 (17)	−0.010 (2)	−0.0078 (18)
C26	0.055 (2)	0.0536 (19)	0.069 (2)	−0.0184 (15)	0.0030 (15)	−0.0143 (15)
C27	0.0378 (16)	0.0525 (17)	0.0474 (14)	−0.0189 (13)	−0.0010 (11)	−0.0094 (12)
C28	0.0545 (18)	0.0442 (16)	0.0440 (15)	0.0014 (14)	−0.0091 (13)	−0.0143 (12)
C29	0.062 (2)	0.0465 (16)	0.0483 (15)	−0.0029 (14)	−0.0081 (14)	−0.0182 (13)
C30	0.093 (3)	0.060 (2)	0.077 (2)	−0.018 (2)	−0.007 (2)	−0.0309 (19)
C30'	0.093 (3)	0.060 (2)	0.077 (2)	−0.018 (2)	−0.007 (2)	−0.0309 (19)
C31	0.127 (4)	0.090 (3)	0.126 (4)	−0.025 (3)	−0.017 (4)	−0.068 (3)
C32	0.166 (6)	0.113 (4)	0.090 (3)	−0.016 (4)	−0.006 (4)	−0.071 (3)
C33	0.141 (5)	0.113 (4)	0.071 (3)	−0.017 (4)	0.020 (3)	−0.052 (3)
C34	0.088 (3)	0.072 (2)	0.060 (2)	−0.009 (2)	0.0062 (19)	−0.0311 (18)
C34'	0.088 (3)	0.072 (2)	0.060 (2)	−0.009 (2)	0.0062 (19)	−0.0311 (18)
C35	0.051 (2)	0.061 (2)	0.0617 (18)	−0.0101 (15)	−0.0041 (15)	−0.0144 (15)
C36	0.076 (2)	0.102 (2)	0.098 (2)	0.0174 (18)	−0.0213 (17)	−0.0365 (17)
C37	0.0629 (17)	0.112 (2)	0.118 (2)	0.0057 (18)	−0.0074 (16)	−0.041 (2)
C38	0.170 (8)	0.118 (5)	0.215 (9)	−0.071 (6)	−0.107 (7)	0.045 (5)
C39	0.272 (12)	0.129 (6)	0.229 (10)	0.046 (7)	−0.139 (9)	−0.079 (7)
C40	0.149 (8)	0.282 (13)	0.184 (9)	−0.041 (8)	−0.018 (7)	0.071 (9)
Cl	0.0475 (5)	0.0849 (6)	0.0735 (5)	−0.0237 (4)	0.0139 (4)	−0.0485 (5)
Mn1	0.0338 (2)	0.0419 (2)	0.0362 (2)	−0.00857 (17)	0.00105 (15)	−0.01302 (17)
N1	0.0364 (13)	0.0580 (14)	0.0446 (12)	−0.0174 (11)	0.0058 (9)	−0.0212 (10)
N2	0.0381 (13)	0.0575 (14)	0.0465 (12)	−0.0189 (11)	0.0002 (10)	−0.0169 (11)
N3	0.0331 (12)	0.0415 (12)	0.0391 (11)	−0.0095 (9)	−0.0003 (8)	−0.0110 (9)
N4	0.0419 (13)	0.0385 (12)	0.0405 (11)	−0.0057 (10)	0.0037 (9)	−0.0073 (9)
N5	0.0388 (13)	0.0437 (12)	0.0411 (11)	−0.0125 (10)	0.0021 (9)	−0.0096 (10)
N6	0.0447 (14)	0.0639 (16)	0.0401 (12)	−0.0200 (12)	0.0080 (10)	−0.0140 (11)
N7	0.0312 (12)	0.0452 (12)	0.0390 (10)	−0.0110 (9)	0.0032 (8)	−0.0146 (9)
N8	0.0567 (15)	0.0949 (19)	0.0960 (17)	0.0071 (14)	−0.0185 (13)	−0.0462 (14)
N9	0.098 (3)	0.084 (3)	0.122 (3)	−0.033 (2)	−0.036 (3)	0.000 (2)
O1	0.0665 (15)	0.0555 (12)	0.0525 (11)	−0.0134 (10)	−0.0004 (10)	−0.0221 (9)
O2	0.0659 (15)	0.0589 (13)	0.0497 (10)	−0.0154 (11)	0.0010 (9)	−0.0249 (10)
O3	0.0425 (13)	0.0660 (14)	0.0571 (12)	0.0012 (10)	−0.0013 (10)	−0.0078 (10)
O4	0.100 (4)	0.090 (4)	0.100 (4)	−0.054 (3)	0.022 (3)	−0.040 (3)
O4'	0.132 (8)	0.133 (8)	0.121 (7)	−0.073 (6)	0.068 (6)	−0.080 (6)
O5	0.096 (2)	0.145 (3)	0.114 (2)	−0.074 (2)	0.0280 (18)	−0.077 (2)
O6	0.124 (3)	0.180 (4)	0.100 (2)	−0.085 (3)	0.046 (2)	−0.098 (2)
O7	0.172 (4)	0.075 (2)	0.094 (2)	−0.025 (2)	0.024 (2)	−0.0268 (17)
O8	0.062 (2)	0.141 (3)	0.145 (3)	0.0034 (19)	0.0054 (19)	−0.054 (3)
O9	0.145 (5)	0.131 (4)	0.347 (9)	−0.087 (4)	−0.056 (5)	0.048 (5)

### Geometric parameters (Å, °)

**Table:**

C1—N7	1.463 (3)	C23—H23	0.9300
C1—C2	1.504 (4)	C24—C25	1.389 (6)
C1—H1A	0.9700	C24—H24	0.9300
C1—H1B	0.9700	C25—C26	1.395 (5)
C2—N1	1.312 (3)	C25—H25	0.9300
C2—N2	1.349 (4)	C26—C27	1.374 (5)
C3—N2	1.462 (4)	C26—H26	0.9300
C3—H3A	0.9600	C27—N5	1.402 (4)
C3—H3B	0.9600	C28—O2	1.254 (4)
C3—H3C	0.9600	C28—O1	1.264 (4)
C4—N2	1.387 (4)	C28—C29	1.483 (4)
C4—C5	1.391 (4)	C29—C34	1.390 (5)
C4—C9	1.392 (4)	C29—C30	1.395 (5)
C5—C6	1.362 (6)	C30—O4	1.288 (6)
C5—H5	0.9300	C30—C31	1.398 (6)
C6—C7	1.402 (6)	C31—C32	1.366 (8)
C6—H6	0.9300	C31—H31	0.9300
C7—C8	1.386 (5)	C32—C33	1.370 (8)
C7—H7	0.9300	C32—H32	0.9300
C8—C9	1.396 (4)	C33—C34	1.384 (6)
C8—H8	0.9300	C33—H33	0.9300
C9—N1	1.405 (4)	C34—H34	0.9300
C10—N7	1.470 (3)	C35—O3	1.211 (4)
C10—C11	1.495 (4)	C35—N8	1.306 (4)
C10—H10A	0.9700	C35—H35	0.9300
C10—H10B	0.9700	C36—N8	1.481 (6)
C11—N3	1.319 (3)	C36—H36A	0.9600
C11—N4	1.348 (3)	C36—H36B	0.9600
C12—N4	1.464 (4)	C36—H36C	0.9600
C12—H12A	0.9600	C37—N8	1.454 (6)
C12—H12B	0.9600	C37—H37A	0.9600
C12—H12C	0.9600	C37—H37B	0.9600
C13—N4	1.385 (4)	C37—H37C	0.9600
C13—C14	1.386 (4)	C38—O9	1.131 (11)
C13—C18	1.400 (4)	C38—N9	1.293 (8)
C14—C15	1.382 (5)	C38—H38	0.9300
C14—H14	0.9300	C39—N9	1.376 (9)
C15—C16	1.387 (5)	C39—H39A	0.9600
C15—H15	0.9300	C39—H39B	0.9600
C16—C17	1.381 (4)	C39—H39C	0.9600
C16—H16	0.9300	C40—N9	1.409 (10)
C17—C18	1.376 (4)	C40—H40A	0.9600
C17—H17	0.9300	C40—H40B	0.9600
C18—N3	1.412 (3)	C40—H40C	0.9600
C19—N7	1.473 (3)	Cl—O8	1.390 (3)
C19—C20	1.485 (4)	Cl—O6	1.400 (3)
C19—H19A	0.9700	Cl—O5	1.400 (3)
C19—H19B	0.9700	Cl—O7	1.444 (3)
C20—N5	1.326 (4)	Mn1—N5	2.228 (2)
C20—N6	1.354 (3)	Mn1—O2	2.286 (2)
C21—N6	1.466 (4)	Mn1—N3	2.297 (2)
C21—H21A	0.9600	Mn1—N1	2.308 (2)
C21—H21B	0.9600	Mn1—O3	2.338 (2)
C21—H21C	0.9600	Mn1—O1	2.358 (2)
C22—N6	1.390 (4)	Mn1—N7	2.502 (2)
C22—C23	1.393 (4)	O4—H4	0.8200
C22—C27	1.395 (4)	O4'—H4'	0.8200
C23—C24	1.365 (6)		
			
N7—C1—C2	108.1 (2)	O4—C30—C29	121.4 (4)
N7—C1—H1A	110.1	O4—C30—C31	119.3 (5)
C2—C1—H1A	110.1	C29—C30—C31	119.2 (5)
N7—C1—H1B	110.1	C32—C31—C30	119.8 (5)
C2—C1—H1B	110.1	C32—C31—H31	120.1
H1A—C1—H1B	108.4	C30—C31—H31	120.1
N1—C2—N2	113.9 (2)	C31—C32—C33	121.5 (4)
N1—C2—C1	122.5 (2)	C31—C32—H32	119.2
N2—C2—C1	123.6 (2)	C33—C32—H32	119.2
N2—C3—H3A	109.5	C32—C33—C34	119.3 (5)
N2—C3—H3B	109.5	C32—C33—H33	120.3
H3A—C3—H3B	109.5	C34—C33—H33	120.3
N2—C3—H3C	109.5	C33—C34—C29	120.5 (5)
H3A—C3—H3C	109.5	C33—C34—H34	119.8
H3B—C3—H3C	109.5	C29—C34—H34	119.8
N2—C4—C5	131.1 (3)	O3—C35—N8	126.7 (3)
N2—C4—C9	106.0 (2)	O3—C35—H35	116.7
C5—C4—C9	122.9 (3)	N8—C35—H35	116.7
C6—C5—C4	116.1 (3)	N8—C36—H36A	109.5
C6—C5—H5	122.0	N8—C36—H36B	109.5
C4—C5—H5	122.0	H36A—C36—H36B	109.5
C5—C6—C7	122.4 (3)	N8—C36—H36C	109.5
C5—C6—H6	118.8	H36A—C36—H36C	109.5
C7—C6—H6	118.8	H36B—C36—H36C	109.5
C8—C7—C6	121.3 (3)	N8—C37—H37A	109.5
C8—C7—H7	119.3	N8—C37—H37B	109.5
C6—C7—H7	119.3	H37A—C37—H37B	109.5
C7—C8—C9	116.9 (3)	N8—C37—H37C	109.5
C7—C8—H8	121.5	H37A—C37—H37C	109.5
C9—C8—H8	121.5	H37B—C37—H37C	109.5
C4—C9—C8	120.3 (3)	O9—C38—N9	128.5 (12)
C4—C9—N1	108.9 (2)	O9—C38—H38	115.7
C8—C9—N1	130.7 (3)	N9—C38—H38	115.8
N7—C10—C11	109.1 (2)	N9—C39—H39A	109.5
N7—C10—H10A	109.9	N9—C39—H39B	109.5
C11—C10—H10A	109.9	H39A—C39—H39B	109.5
N7—C10—H10B	109.9	N9—C39—H39C	109.5
C11—C10—H10B	109.9	H39A—C39—H39C	109.5
H10A—C10—H10B	108.3	H39B—C39—H39C	109.5
N3—C11—N4	113.7 (2)	N9—C40—H40A	109.5
N3—C11—C10	123.5 (2)	N9—C40—H40B	109.5
N4—C11—C10	122.8 (2)	H40A—C40—H40B	109.5
N4—C12—H12A	109.5	N9—C40—H40C	109.5
N4—C12—H12B	109.5	H40A—C40—H40C	109.5
H12A—C12—H12B	109.5	H40B—C40—H40C	109.5
N4—C12—H12C	109.5	O8—Cl—O6	115.7 (2)
H12A—C12—H12C	109.5	O8—Cl—O5	111.7 (2)
H12B—C12—H12C	109.5	O6—Cl—O5	110.3 (2)
N4—C13—C14	131.1 (3)	O8—Cl—O7	103.8 (2)
N4—C13—C18	106.1 (2)	O6—Cl—O7	106.7 (2)
C14—C13—C18	122.8 (3)	O5—Cl—O7	108.0 (2)
C15—C14—C13	116.5 (3)	N5—Mn1—O2	144.47 (8)
C15—C14—H14	121.8	N5—Mn1—N3	103.75 (8)
C13—C14—H14	121.8	O2—Mn1—N3	93.62 (8)
C14—C15—C16	121.0 (3)	N5—Mn1—N1	118.65 (8)
C14—C15—H15	119.5	O2—Mn1—N1	86.93 (8)
C16—C15—H15	119.5	N3—Mn1—N1	101.58 (8)
C17—C16—C15	122.1 (3)	N5—Mn1—O3	78.95 (8)
C17—C16—H16	119.0	O2—Mn1—O3	81.04 (8)
C15—C16—H16	119.0	N3—Mn1—O3	173.46 (8)
C18—C17—C16	117.8 (3)	N1—Mn1—O3	81.99 (9)
C18—C17—H17	121.1	N5—Mn1—O1	92.95 (8)
C16—C17—H17	121.1	O2—Mn1—O1	56.05 (8)
C17—C18—C13	119.7 (2)	N3—Mn1—O1	89.21 (8)
C17—C18—N3	131.7 (3)	N1—Mn1—O1	142.21 (8)
C13—C18—N3	108.6 (2)	O3—Mn1—O1	84.68 (8)
N7—C19—C20	109.3 (2)	N5—Mn1—N7	70.46 (8)
N7—C19—H19A	109.8	O2—Mn1—N7	145.07 (8)
C20—C19—H19A	109.8	N3—Mn1—N7	69.47 (7)
N7—C19—H19B	109.8	N1—Mn1—N7	68.01 (8)
C20—C19—H19B	109.8	O3—Mn1—N7	117.06 (8)
H19A—C19—H19B	108.3	O1—Mn1—N7	147.91 (8)
N5—C20—N6	113.1 (2)	C2—N1—C9	104.7 (2)
N5—C20—C19	121.9 (2)	C2—N1—Mn1	113.35 (18)
N6—C20—C19	125.0 (2)	C9—N1—Mn1	135.86 (19)
N6—C21—H21A	109.5	C2—N2—C4	106.4 (2)
N6—C21—H21B	109.5	C2—N2—C3	128.0 (3)
H21A—C21—H21B	109.5	C4—N2—C3	125.5 (3)
N6—C21—H21C	109.5	C11—N3—C18	104.8 (2)
H21A—C21—H21C	109.5	C11—N3—Mn1	115.48 (17)
H21B—C21—H21C	109.5	C18—N3—Mn1	136.64 (18)
N6—C22—C23	132.0 (3)	C11—N4—C13	106.9 (2)
N6—C22—C27	105.8 (2)	C11—N4—C12	127.0 (3)
C23—C22—C27	122.3 (3)	C13—N4—C12	126.1 (2)
C24—C23—C22	116.6 (4)	C20—N5—C27	105.0 (2)
C24—C23—H23	121.7	C20—N5—Mn1	119.63 (17)
C22—C23—H23	121.7	C27—N5—Mn1	135.29 (19)
C23—C24—C25	121.9 (3)	C20—N6—C22	107.0 (2)
C23—C24—H24	119.0	C20—N6—C21	126.9 (3)
C25—C24—H24	119.0	C22—N6—C21	126.1 (3)
C24—C25—C26	121.3 (4)	C1—N7—C10	111.3 (2)
C24—C25—H25	119.4	C1—N7—C19	111.7 (2)
C26—C25—H25	119.4	C10—N7—C19	110.9 (2)
C27—C26—C25	117.4 (3)	C1—N7—Mn1	106.10 (16)
C27—C26—H26	121.3	C10—N7—Mn1	108.06 (15)
C25—C26—H26	121.3	C19—N7—Mn1	108.56 (16)
C26—C27—C22	120.5 (3)	C35—N8—C37	123.1 (4)
C26—C27—N5	130.3 (3)	C35—N8—C36	119.6 (4)
C22—C27—N5	109.3 (3)	C37—N8—C36	117.3 (3)
O2—C28—O1	120.1 (3)	C38—N9—C39	128.2 (10)
O2—C28—C29	120.1 (3)	C38—N9—C40	117.3 (9)
O1—C28—C29	119.8 (3)	C39—N9—C40	114.6 (8)
C34—C29—C30	119.5 (3)	C28—O1—Mn1	90.11 (18)
C34—C29—C28	120.6 (3)	C28—O2—Mn1	93.71 (18)
C30—C29—C28	119.9 (3)	C35—O3—Mn1	127.9 (2)
			
N7—C1—C2—N1	−13.4 (4)	N5—Mn1—N3—C18	−115.2 (2)
N7—C1—C2—N2	165.4 (2)	O2—Mn1—N3—C18	33.6 (2)
N2—C4—C5—C6	−178.3 (4)	N1—Mn1—N3—C18	121.2 (2)
C9—C4—C5—C6	1.2 (5)	O3—Mn1—N3—C18	−1.4 (8)
C4—C5—C6—C7	−0.5 (7)	O1—Mn1—N3—C18	−22.3 (2)
C5—C6—C7—C8	0.3 (7)	N7—Mn1—N3—C18	−177.8 (3)
C6—C7—C8—C9	−0.8 (6)	N3—C11—N4—C13	0.3 (3)
N2—C4—C9—C8	177.9 (3)	C10—C11—N4—C13	179.2 (2)
C5—C4—C9—C8	−1.7 (5)	N3—C11—N4—C12	179.3 (3)
N2—C4—C9—N1	−0.4 (3)	C10—C11—N4—C12	−1.8 (4)
C5—C4—C9—N1	−180.0 (3)	C14—C13—N4—C11	−178.0 (3)
C7—C8—C9—C4	1.4 (5)	C18—C13—N4—C11	0.3 (3)
C7—C8—C9—N1	179.2 (3)	C14—C13—N4—C12	3.0 (5)
N7—C10—C11—N3	−15.4 (4)	C18—C13—N4—C12	−178.7 (3)
N7—C10—C11—N4	165.7 (2)	N6—C20—N5—C27	0.6 (3)
N4—C13—C14—C15	179.3 (3)	C19—C20—N5—C27	−177.1 (2)
C18—C13—C14—C15	1.3 (4)	N6—C20—N5—Mn1	177.20 (17)
C13—C14—C15—C16	−0.2 (4)	C19—C20—N5—Mn1	−0.4 (3)
C14—C15—C16—C17	0.1 (5)	C26—C27—N5—C20	179.7 (3)
C15—C16—C17—C18	−1.0 (5)	C22—C27—N5—C20	−0.1 (3)
C16—C17—C18—C13	2.1 (4)	C26—C27—N5—Mn1	3.8 (5)
C16—C17—C18—N3	−178.0 (3)	C22—C27—N5—Mn1	−175.91 (19)
N4—C13—C18—C17	179.3 (2)	O2—Mn1—N5—C20	−164.32 (18)
C14—C13—C18—C17	−2.3 (4)	N3—Mn1—N5—C20	−47.3 (2)
N4—C13—C18—N3	−0.7 (3)	N1—Mn1—N5—C20	64.4 (2)
C14—C13—C18—N3	177.8 (3)	O3—Mn1—N5—C20	138.8 (2)
N7—C19—C20—N5	−24.6 (3)	O1—Mn1—N5—C20	−137.2 (2)
N7—C19—C20—N6	158.0 (2)	N7—Mn1—N5—C20	14.67 (19)
N6—C22—C23—C24	−179.4 (3)	O2—Mn1—N5—C27	11.1 (3)
C27—C22—C23—C24	0.3 (5)	N3—Mn1—N5—C27	128.1 (2)
C22—C23—C24—C25	−0.4 (5)	N1—Mn1—N5—C27	−120.2 (2)
C23—C24—C25—C26	0.2 (6)	O3—Mn1—N5—C27	−45.8 (2)
C24—C25—C26—C27	0.2 (5)	O1—Mn1—N5—C27	38.2 (3)
C25—C26—C27—C22	−0.4 (5)	N7—Mn1—N5—C27	−170.0 (3)
C25—C26—C27—N5	179.9 (3)	N5—C20—N6—C22	−0.8 (3)
N6—C22—C27—C26	179.8 (3)	C19—C20—N6—C22	176.7 (3)
C23—C22—C27—C26	0.1 (5)	N5—C20—N6—C21	−178.4 (3)
N6—C22—C27—N5	−0.4 (3)	C19—C20—N6—C21	−0.9 (5)
C23—C22—C27—N5	179.9 (3)	C23—C22—N6—C20	−179.6 (3)
O2—C28—C29—C34	10.5 (5)	C27—C22—N6—C20	0.7 (3)
O1—C28—C29—C34	−171.4 (3)	C23—C22—N6—C21	−1.9 (5)
O2—C28—C29—C30	−168.7 (3)	C27—C22—N6—C21	178.4 (3)
O1—C28—C29—C30	9.4 (5)	C2—C1—N7—C10	−77.4 (3)
C34—C29—C30—O4	175.3 (5)	C2—C1—N7—C19	158.1 (2)
C28—C29—C30—O4	−5.5 (6)	C2—C1—N7—Mn1	40.0 (2)
C34—C29—C30—C31	−2.9 (6)	C11—C10—N7—C1	151.1 (2)
C28—C29—C30—C31	176.3 (4)	C11—C10—N7—C19	−83.9 (3)
O4—C30—C31—C32	−175.6 (6)	C11—C10—N7—Mn1	34.9 (2)
C29—C30—C31—C32	2.6 (7)	C20—C19—N7—C1	−83.4 (3)
C30—C31—C32—C33	0.0 (9)	C20—C19—N7—C10	151.8 (2)
C31—C32—C33—C34	−2.3 (9)	C20—C19—N7—Mn1	33.2 (2)
C32—C33—C34—C29	1.9 (7)	N5—Mn1—N7—C1	94.11 (16)
C30—C29—C34—C33	0.7 (6)	O2—Mn1—N7—C1	−86.9 (2)
C28—C29—C34—C33	−178.6 (4)	N3—Mn1—N7—C1	−152.17 (17)
N2—C2—N1—C9	−1.1 (3)	N1—Mn1—N7—C1	−39.69 (16)
C1—C2—N1—C9	177.9 (3)	O3—Mn1—N7—C1	28.29 (18)
N2—C2—N1—Mn1	156.12 (19)	O1—Mn1—N7—C1	156.51 (16)
C1—C2—N1—Mn1	−24.9 (3)	N5—Mn1—N7—C10	−146.41 (18)
C4—C9—N1—C2	0.9 (3)	O2—Mn1—N7—C10	32.6 (2)
C8—C9—N1—C2	−177.2 (3)	N3—Mn1—N7—C10	−32.69 (16)
C4—C9—N1—Mn1	−148.4 (2)	N1—Mn1—N7—C10	79.79 (17)
C8—C9—N1—Mn1	33.6 (5)	O3—Mn1—N7—C10	147.77 (16)
N5—Mn1—N1—C2	−16.9 (2)	O1—Mn1—N7—C10	−84.0 (2)
O2—Mn1—N1—C2	−171.0 (2)	N5—Mn1—N7—C19	−26.04 (16)
N3—Mn1—N1—C2	96.0 (2)	O2—Mn1—N7—C19	152.94 (16)
O3—Mn1—N1—C2	−89.6 (2)	N3—Mn1—N7—C19	87.69 (17)
O1—Mn1—N1—C2	−160.06 (17)	N1—Mn1—N7—C19	−159.83 (18)
N7—Mn1—N1—C2	33.94 (19)	O3—Mn1—N7—C19	−91.85 (17)
N5—Mn1—N1—C9	130.5 (2)	O1—Mn1—N7—C19	36.4 (2)
O2—Mn1—N1—C9	−23.5 (3)	O3—C35—N8—C37	−178.0 (4)
N3—Mn1—N1—C9	−116.6 (3)	O3—C35—N8—C36	4.8 (6)
O3—Mn1—N1—C9	57.8 (3)	O9—C38—N9—C39	−0.2 (12)
O1—Mn1—N1—C9	−12.6 (3)	O9—C38—N9—C40	179.0 (8)
N7—Mn1—N1—C9	−178.6 (3)	O2—C28—O1—Mn1	1.5 (3)
N1—C2—N2—C4	0.9 (3)	C29—C28—O1—Mn1	−176.6 (2)
C1—C2—N2—C4	−178.1 (3)	N5—Mn1—O1—C28	−162.24 (17)
N1—C2—N2—C3	178.3 (3)	O2—Mn1—O1—C28	−0.87 (16)
C1—C2—N2—C3	−0.6 (5)	N3—Mn1—O1—C28	94.03 (17)
C5—C4—N2—C2	179.3 (3)	N1—Mn1—O1—C28	−14.0 (2)
C9—C4—N2—C2	−0.2 (3)	O3—Mn1—O1—C28	−83.63 (17)
C5—C4—N2—C3	1.7 (6)	N7—Mn1—O1—C28	141.01 (17)
C9—C4—N2—C3	−177.8 (3)	O1—C28—O2—Mn1	−1.6 (3)
N4—C11—N3—C18	−0.7 (3)	C29—C28—O2—Mn1	176.5 (2)
C10—C11—N3—C18	−179.7 (2)	N5—Mn1—O2—C28	34.2 (2)
N4—C11—N3—Mn1	162.72 (17)	N3—Mn1—O2—C28	−85.73 (18)
C10—C11—N3—Mn1	−16.2 (3)	N1—Mn1—O2—C28	172.85 (18)
C17—C18—N3—C11	−179.1 (3)	O3—Mn1—O2—C28	90.48 (18)
C13—C18—N3—C11	0.9 (3)	O1—Mn1—O2—C28	0.88 (16)
C17—C18—N3—Mn1	22.9 (4)	N7—Mn1—O2—C28	−144.18 (17)
C13—C18—N3—Mn1	−157.14 (19)	N8—C35—O3—Mn1	177.4 (3)
N5—Mn1—N3—C11	88.48 (18)	N5—Mn1—O3—C35	−172.7 (3)
O2—Mn1—N3—C11	−122.76 (18)	O2—Mn1—O3—C35	36.8 (3)
N1—Mn1—N3—C11	−35.17 (19)	N3—Mn1—O3—C35	72.2 (8)
O3—Mn1—N3—C11	−157.7 (6)	N1—Mn1—O3—C35	−51.3 (3)
O1—Mn1—N3—C11	−178.67 (18)	O1—Mn1—O3—C35	93.3 (3)
N7—Mn1—N3—C11	25.83 (17)	N7—Mn1—O3—C35	−111.5 (3)

### Hydrogen-bond geometry (Å, °)

**Table:**

*D*—H···*A*	*D*—H	H···*A*	*D*···*A*	*D*—H···*A*
O4'—H4'···O2	0.82	1.82	2.554 (8)	149
O4—H4···O1	0.82	1.79	2.530 (5)	149
